# Effects of red blood cell transfusion on hemodynamic parameters: a prospective study in intensive care unit patients

**DOI:** 10.1186/1757-7241-21-21

**Published:** 2013-03-25

**Authors:** Bernd Saugel, Michaela Klein, Alexander Hapfelmeier, Veit Phillip, Caroline Schultheiss, Agnes S Meidert, Marlena Messer, Roland M Schmid, Wolfgang Huber

**Affiliations:** 1 II. Medizinische Klinik und Poliklinik, Klinikum rechts der Isar der Technischen Universität München, Ismaninger Strasse 22, München 81675, Germany; 2 Institut für Medizinische Statistik und Epidemiologie, Klinikum rechts der Isar der Technischen Universität München, Ismaninger Strasse 22, München, 81675, Germany

**Keywords:** Red blood cells, Transfusion, Transpulmonary thermodilution, Hemodynamic monitoring, Critical care

## Abstract

**Background:**

The aim of the study was to investigate the effect of red blood cell (RBC) transfusion on hemodynamic parameters including transpulmonary thermodilution (TPTD)-derived variables.

**Methods:**

We compared hemodynamic parameters obtained before and after RBC transfusion (2 RBC units) in 34 intensive care unit (ICU) patients.

**Results:**

Directly after RBC transfusion, we observed a significant increase in hematocrit (28 ± 3 vs. 22 ± 2%, p < 0.001), hemoglobin (9.4 ± 0.9 vs. 7.6 ± 0.8 g/dL, p < 0.001), arterial oxygen content (CaO_2_) (12.2 ± 1.2 vs. 9.9 ± 1.0 mL/dL, p < 0.001), and oxygen delivery (DO_2_) (1073 ± 369 vs. 934 ± 288 mL/min, p < 0.001) compared with baseline. Cardiac output (CO) (8.89 ± 3.06 vs. 9.42 ± 2.75 L/min, p = 0.020), cardiac index (CI) (4.53 ± 1.36 vs. 4.82 ± 1.21 L/min/m^2^, p = 0.016), and heart rate (91 ± 16 vs. 95 ± 14 bpm, p = 0.007) were significantly lower following RBC transfusion while no significant change in stroke volume (SV) was observed. Mean arterial pressure (MAP) (median 87 vs. 78 mmHg, p < 0.001) and systemic vascular resistance index (SVRI) (median 1212 vs. 1103 dyn*s*cm^-5^*m^2^, p = 0.001) significantly increased directly after RBC transfusion. Global end-diastolic volume index (GEDVI), extravascular lung water index (EVLWI), and pulmonary vascular permeability index (PVPI) did not significantly change.

**Conclusions:**

In ICU patients, the transfusion of 2 RBC units induces a significant decrease in CO and CI because of a significant decrease in heart rate (while SV remains unchanged). Despite the decrease in CO, DO_2_ significantly increases because of a significant increase in CaO_2_. In addition, RBC transfusion results in a significant increase in MAP and SVRI. No significant changes in TPTD-parameters reflecting cardiac preload (GEDVI), pulmonary edema (EVLWI), and pulmonary vascular permeability (PVPI) are observed following RBC transfusion.

## Background

Anemia is a common finding in critically ill patients treated in the intensive care unit (ICU) [1]. Because anemia has been shown to be associated with increased morbidity and mortality [2-4], it is usually treated with red blood cell (RBC) transfusion. However, also RBC transfusions have been demonstrated to be associated with organ failure and increased mortality [1,4]. Current clinical practice guidelines for RBC transfusion therefore recommend a restrictive transfusion strategy (with a hemoglobin level of 7 to 8 g/dL used as a transfusion trigger) in hospitalized, stable patients [5]. However, there is still a considerable debate regarding risks and benefits of and indications for RBC transfusion, especially in critically ill patients [6]. Because there are very limited data on the impact of RBC transfusion on advanced hemodynamic parameters reflecting cardiopulmonary function, it was the aim of our study to investigate the effects of RBC transfusion on hemodynamic variables obtained using single-indicator transpulmonary thermodilution (TPTD) in ICU patients.

## Methods

### Study design and patients

This was a prospective study in ICU patients receiving RBC transfusions in a German university hospital. Patients (18 years of age or older) monitored using TPTD who received 2 units of RBC concentrates for treatment of anemia not related to acute hemorrhage were eligible for study inclusion. The indication for RBC transfusion was made independently from the study by the ICU physician in charge. During the study period, usually a hemoglobin value of about 7 g/dL was used as a transfusion trigger in our ICU. According to our ICU standard operating procedures, vasoactive agents were titrated to maintain a mean arterial pressure (MAP) above 65 mmHg. In patients included in the study, TPTD measurements were performed immediately before, directly after, and 2 hours after RBC transfusion. In parallel, data regarding respiratory function (arterial blood gas analysis and ventilator settings), central venous oxygen saturation (ScvO_2_), and basic hemodynamic data such as systolic and diastolic blood pressure, MAP, heart rate, and central venous pressure (CVP) were recorded. Thirty-five patients were included in the study. One patient was not included in the statistical analysis because the central venous catheter was inserted via the femoral vein thus not allowing accurate determination of ScvO_2_, CVP, and TPTD-based cardiac preload parameters [7,8].

The study was approved by the institutional review board (Ethikkommission der Fakultät für Medizin der Technischen Universität München) and written informed consent was obtained from all patients or their legal representatives.

### Arterial oxygen content and oxygen delivery

Arterial oxygen content (CaO_2_) was calculated as follows:

$$\begin{array}{l} {\text{CaO}}_{2}\;\left[\mathrm{mL}/\mathrm{dL}\right]\;=\;\left(1.34\;\left[\mathrm{mL}/\text{g}\right]\right)\;\times \;\mathrm{Hb}\;\left[\text{g}/\mathrm{dl}\right]\;\\ \;\times \;\left({\mathrm{SaO}}_{2}\;\left[\text{expressed}\;\mathrm{as}\;\text{a}\;\text{fraction}\;\mathrm{of}\;1.0\right]\right)\;\\ \;+\;\left(0.0031\;\left[\mathrm{mL}/\mathrm{dL}/\text{mmHg}\right]\right)\;\\ \;\times \;\left({\text{PaO}}_{2}\;\left[\text{mmHg}\right]\right) \end{array}$$

(1.34, Hüfner’s constant; Hb, hemoglobin concentration; SaO_2_, arterial oxygen saturation; 0.0031, oxygen solubility coefficient; PaO_2_, arterial partial pressure of oxygen)

Oxygen delivery (DO_2_) was obtained using the formula:

$$\begin{array}{l} {\mathrm{DO}}_{2}\;\left[\mathrm{mL}/\text{min}\right]\;=\;\mathrm{CO}\;\left[\text{L}/\text{min}\right]\;\\ \;\times \;{\text{CaO}}_{2}\;\left[\mathrm{mL}/\mathrm{dL}\right]\;\times \;10 \end{array}$$

(CO, cardiac output)

### Measurement of hemodynamic parameters

Cardiac index (CI), global end-diastolic volume index (GEDVI), systemic vascular resistance index (SVRI), extravascular lung water index (EVLWI), pulmonary vascular permeability index (PVPI), and index of left ventricular contractility (dPmax) were determined using single-indicator TPTD [9-17]. TPTD measurements were performed in triplicate as described before [18] using a 5-French femoral arterial catheter (Pulsiocath, Pulsion Medical Systems, Munich, Germany) and a PiCCOplus or PiCCO2 monitor (Pulsion Medical Systems). In 1 patient, PVPI values were missing. CI was obtained by indexation of cardiac output (CO) to body surface area. Stroke volume (SV) was calculated by dividing CO by heart rate. Cardiac power index (CPI) was calculated as follows: CPI = MAP × CI × 0.0022.

### Statistical analysis

IBM SPSS Statistics 20 (SPSS inc., Chicago, IL, USA) was used for all statistical analyses in this study. To present descriptive statistics, we calculated mean ± standard deviation for normally distributed continuous data, median and 25%-75% percentile range (i.e. interquartile range) for not normally distributed continuous data, and absolute and relative frequencies for categorical data. To compare the hemodynamic variables before and after RBC transfusion (primary endpoint) as well as before and 2 hours after RBC transfusion, we performed the *t*-test for paired samples and the Wilcoxon signed rank test for paired samples for normally distributed data and not normally distributed data, respectively. A p-value below a significance level of 5% (p < 0.05) indicates statistical significance.

## Results

### Patients’ characteristics

Thirty-four patients who received RBC transfusions (each RBC transfusion consisting of 2 units of RBC concentrates) were included in the final analysis. The demographic and clinical characteristics of these patients are presented in Table 1.

**Table 1:** Patients


**Patients’ characteristics**	
Sex, male, n (%)	22 (65%)
Age, years	59 ± 13
Height, cm	172 ± 8
Body weight, kg	78 (74-90)
**Reason for intensive care unit treatment**	
Liver cirrhosis/acute liver failure, n (%)	13 (38%)
Acute respiratory insufficiency/pneumonia, n (%)	11 (32%)
Severe sepsis/septic multiple organ dysfunction syndrome, n (%)	6 (18%)
Cardiopulmonary resuscitation, n (%)	2 (6%)
Other, n (%)	2 (6%)
**Clinical characteristics on day of red blood cell transfusion**	
Therapeutic Intervention Scoring System, points	22 ± 9
Mechanical ventilation, n (%)	23 (68%)
Norepinephrine therapy, n (%)	13 (38%)

Data are presented as absolute and relative frequencies (categorical data), mean ± standard deviation (normally distributed continuous data), or median and 25%-75% percentile range (not normally distributed continuous data).

### Effect of red blood cell transfusion on hematocrit, hemoglobin, arterial oxygen content, oxygen delivery, arterial blood gas analysis, and central venous oxygen saturation

Compared with baseline, RBC transfusion resulted in a statistically significant increase in hematocrit, hemoglobin, CaO_2_, and DO_2_ when determined directly after and 2 hours after the transfusion (Table 2). PaO_2_, arterial partial pressure of carbon dioxide (PaCO_2_), SaO_2_, and ScvO_2_ were not statistically significantly different before and after RBC transfusion.

**Table 2:** Effects of red blood cell transfusion on hematocrit, hemoglobin, arterial oxygen content, oxygen delivery, arterial blood gas analysis, and central venous oxygen saturation

**Parameter**	**Before RBC transfusion**	**After RBC transfusion**	**p-value compared with baseline**	**2 h after RBC transfusion**	**p-value compared with baseline**
Hematocrit, %	22 ± 2	28 ± 3	**<0.001**	27 ± 2	**<0.001**
Hemoglobin, g/dL	7.6 ± 0.8	9.4 ± 0.9	**<0.001**	9.2 ± 0.8	**<0.001**
CaO_2_, mL/dL	9.9 ± 1.0	12.2 ± 1.2	**<0.001**	12.0 ± 1.1	**<0.001**
DO_2_, mL/min	934 ± 288	1073 ± 369	**<0.001**	1042 ± 334	**0.002**
PaO_2_, mmHg	84.0 ± 13.1	82.7 ± 16.8	0.700	89.1 ± 18.4	0.227
PaCO_2_, mmHg	48.3 ± 11.7	48.6 ± 13.1	0.785	48.4 ± 14.3	0.972
SaO_2_, %	95.4 (93.1-96.8)	95.5 (92.4-96.7)	0.650	95.5 (94.2-97.1)	0.543
ScvO2, %	71.6 ± 6.2	71.1 ± 7.1	0.581	73.9 ± 7.3	0.113

*RBC *red blood cell, *DO*_*2 *_oxygen delivery, *CaO*_*2 *_arterial oxygen content, *PaO*_*2 *_arterial partial pressure of oxygen, *PaCO*_*2 *_arterial partial pressure of carbon dioxide, *SaO*_*2 *_arterial oxygen saturation, *ScvO2 *central venous oxygen saturation. Data are presented as mean ± standard deviation (normally distributed data) or median and 25%-75% percentile range (not normally distributed data).

### Effects of red blood cell transfusion on hemodynamics

The effects of RBC transfusion on hemodynamic variables measured directly after and 2 hours after the transfusion are presented in Table 3.

**Table 3:** Effects of red blood cell transfusion on hemodynamic variables

**Parameter**	**Before RBC transfusion**	**After RBC transfusion**	**p-value compared with baseline**	**2 h after RBC transfusion**	**p-value compared with baseline**
**Basic hemodynamic parameters**					
Heart rate, bpm	95 ± 14	91 ± 16	**0.007**	90 ± 16	**0.012**
Systolic blood pressure, mmHg	130 (114-150)	136 (126-150)	**0.001**	131 (117-144)	0.852
Diastolic blood pressure, mmHg	57 (52-65)	63 (60-69)	**<0.001**	60 (55-67)	0.102
MAP, mmHg	78 (74-93)	87 (84-97)	**<0.001**	84 (77-93)	0.176
**Cardiac preload**					
CVP, mmHg	16 ± 7	17 ± 7	**0.048**	16 ± 7	0.514
GEDVI, mL/m^2^	882 ± 238	869 ± 228	0.304	874 ± 223	0.626
**Cardiac function**					
CO, L/min	9.42 ± 2.75	8.89 ± 3.06	**0.020**	8.70 ± 2.75	**0.005**
CI, L/min/m^2^	4.82 ± 1.21	4.53 ± 1.36	**0.016**	4.45 ± 1.28	**0.004**
SV, mL	100 ± 28	99 ± 30	0.512	98 ± 30	0.487
CPI, W/m^2^	0.89 ± 0.28	0.91 ± 0.27	0.403	0.85 ± 0.28	0.268
dPmax, mmHg/s	1394 (1136-1726)	1414 (1200-1677)	0.851	1287 (1056-1662)	0.245
**Vascular resistance**					
SVRI, dyn*s*cm^-5^*m^2^	1103 (902-1422)	1212 (1028-1856)	**0.001**	1193 (951-1612)	**0.010**
**Pulmonary hydration and permeability**					
EVLWI, mL/kg	11 (9-14)	10 (9-14)	0.283	10 (8-13)	0.286
PVPI	1.7 (1.4-2.5)	1.8 (1.5-2.4)	0.243	1.7 (1.4-2.4)	0.992

*MAP *mean arterial pressure, *CVP *central venous pressure, *GEDVI* global end-diastolic volume index, *CI *cardiac index, *CO* cardiac output, *SV *stroke volume, *CPI *cardiac power index, *dPmax *index of left ventricular contractility, *SVRI *systemic vascular resistance index, *EVLWI *extravascular lung water index, *PVPI* pulmonary vascular permeability index. Data are presented as mean ± standard deviation (normally distributed data) or median and 25%-75% percentile range (not normally distributed data).

CO and CI were statistically significantly lower directly after and 2 hours after RBC transfusion compared with baseline. Since there was no statistically significant RBC transfusion-induced change in SV, the decrease in CO/CI is explainable by the significant decrease in heart rate. Whereas MAP and SVRI significantly increased directly after RBC transfusion, the TPTD-derived cardiac preload parameter GEDVI did not significantly change. Following RBC transfusion, there was no significant change in CPI and dPmax compared with baseline values. In addition, RBC transfusions did not significantly alter EVLWI or PVPI. In patients with the need for norepinephrine therapy, the vasopressor dose was lower after RBC transfusion compared with baseline (baseline: 0.07 (0.03-0.10) μg/kg/min, after RBC transfusion: 0.05 (0.02-0.08) μg/kg/min, p = 0.051).

## Discussion

In this study, we evaluated the effects of RBC transfusion on advanced parameters of cardiopulmonary function obtained using TPTD in ICU patients. Following the transfusion of 2 units of RBC concentrates, CO and CI were significantly lower compared with baseline values, because RBC transfusion resulted in a significant decrease in heart rate while no statistically significant change in SV was observed. Despite the decrease in CO, we observed a significant increase in DO_2_ due to a significant increase in CaO_2_. Whereas MAP and SVRI significantly increased directly after RBC transfusion, there was no statistically significant change in CPI, dPmax, and the TPTD-derived cardiac preload parameter GEDVI compared with baseline values. No significant changes in parameters reflecting pulmonary edema (EVLWI) and pulmonary vascular permeability (PVPI) were observed following RBC transfusion.

The finding that CO/CI are significantly lower following RBC transfusion might be explained as follows. Our results demonstrate that the increase in hemoglobin concentration following RBC transfusion results in an increase in CaO_2_ as well as in an increase of MAP and SVRI. The RBC transfusion-associated improvement of tissue oxygenation (i.e. increase in CaO_2_) might attenuate the anemia/hypoxemia-induced sympathoadrenal response [19] and might therefore result in a decrease in heart rate in patients treated with RBC transfusion. In parallel with the decrease in heart rate, CO/CI also decreases because SV remains unchanged after RBC transfusion. Since MAP increases in parallel with a decrease in heart rate, no change in CPI can be observed when measured directly after RBC transfusion.

In our study, we recorded hemodynamic data before, directly after, and additionally 2 hours after RBC transfusion. Interestingly, while arterial pressure values (MAP, systolic and diastolic blood pressure) were statistically significantly higher when determined directly after RBC transfusion compared with baseline values but not when measured 2 hours after the transfusion, the RBC transfusion-associated effects on CO/CI were even more pronounced 2 hours after than directly after transfusion. This finding demonstrates that the observed effects on cardiac function are not only short-term effects (e.g., resulting from a volume resuscitation effect of RBC transfusion), but are the result of complex RBC transfusion-induced changes of central hemodynamic parameters.

At first glance, it is surprising that we did not observe a significant increase in the cardiac preload parameter GEDVI following transfusion of 2 RBC units. In addition, the statistically significant increase in CVP (16 ± 7 to 17 ± 7 mmHg) seems to be of very limited clinical relevance. However, patients included in the trial were in a state of isovolemic anemia, because anemia due to acute hemorrhage was an exclusion criterion in our study. Furthermore, patients included in the study had in large part been treated in the ICU for a longer time period before study inclusion (including volume resuscitation guided by advanced hemodynamic monitoring using TPTD). Since the hematocrit level markedly and significantly increased following RBC transfusion whereas cardiac preload parameters remained unchanged, we hypothesize that in our patients a part of the additional blood volume was shifted from the central circulation to capacity vessels.

Considering the fact that SVRI is not a directly measured but a calculated parameter (derived from the formula SVRI = (MAP – CVP)/CI x constant), the finding that SVRI increased following RBC transfusion is a logical mathematical result associated with the observed changes in MAP, CVP and CI.

Despite the significant increase in CaO_2_ and DO_2_, ScvO_2_ remained unchanged following RBC transfusion compared with baseline values in our study. Considering previous data [20], one might speculate that a reduction in oxygen extraction following RBC transfusion can explain the finding that ScvO_2_ did not increase after RBC transfusion. However, to definitely interpret this finding, one would need to exactly know oxygen extraction (and therefore oxygen consumption) in addition to DO_2_ before and after RBC transfusion. Unfortunately, we are not able to calculate oxygen consumption because we did not use a pulmonary artery catheter for hemodynamic measurements in our study and therefore are unable to report mixed venous oxygen saturation (SvO_2_) values. Therefore, although ScvO_2_ might be used as a surrogate marker for SvO_2_, we are not able to draw definite conclusions about oxygen extraction and the finding that ScvO_2_ was not influenced by RBC transfusion.

In a previous study [20], Gramm et al. studied the hemodynamic effects of RBC transfusion in 19 patients using a pulmonary artery catheter. In contrast, we used TPTD to determine hemodynamic variables in our trial. Besides this, several differences between our study and the study of Gramm et al. regarding the study design as well as the results need to be mentioned. First, Gramm and colleagues studied 19 mechanically ventilated surgical patients with sepsis, whereas we investigated a mixed and unselected ICU population. In accordance with our results, Gramm et al. observed a significant increase in CaO_2_ and a significant decrease in heart rate. Furthermore, DO_2_ also markedly increased following RBC transfusion in their study. However, in contrast to our data, this increase in DO_2_ was not statistically significant. In addition, contrary to our findings (demonstrating a RBC transfusion-induced decrease in CO and CI), CO and CI were not significantly different after transfusion of RBC in the study by Gramm and colleagues. This might be due to the fact that they solely included septic patients in whom a hyperdynamic hemodynamic state (with high CO) can regularly be observed.

Transfusion-related acute lung injury (TRALI) has been described as an important and severe adverse effect of RBC transfusion in critically ill patients [21-24]. In TRALI, the pulmonary vascular permeability is increased following transfusion of blood products resulting in pulmonary edema not associated with other factors like sepsis, volume overload, or heart insufficiency [21]. Because of the limited number of patients included in our trial and because of the fact that the patients received only 2 units of RBC concentrates each, rigorous conclusions about TRALI cannot be drawn from our study. However, according to TPTD-derived parameters, in our study, there was no sign for transfusion-related pulmonary fluid overload, because no statistically significant changes in TPTD-derived markers of pulmonary edema (EVLWI) and pulmonary vascular permeability (PVPI) were observed following RBC transfusion.

For TPTD measurements, we used the PiCCO technology in our study. This technology for advanced hemodynamic monitoring allows the determination of various cardiopulmonary parameters (among others CI, GEDVI, SVRI, EVLWI, PVPI, and dPmax) based on the principles of TPTD and pulse contour analysis [9-17,25]. Therefore, TPTD can be used to assess a patient’s cardiac function, cardiac preload, and pulmonary fluid status [18]. Although the PiCCO system is less invasive compared to a pulmonary artery catheter, the TPTD technology requires the insertion of a central venous catheter and the placement of an arterial catheter in the abdominal aorta through the femoral artery [25,26]. In addition, some further limitations of the technology have to be mentioned. For accurate determination of pulse contour analysis-derived hemodynamic variables, the system needs to be re-calibrated regularly by using TPTD [27]. Valvulopathies can influence and adulterate TPTD-derived volumetric cardiac preload variables [28]. Furthermore, there are data indicating that the TPTD technology might be further improved by using a more differentiated view regarding the definition of normal ranges and regarding the indexation of TPTD-variables to biometric parameters [29-31].

The results of our study contribute to the understanding of the effects of RBC transfusion on hemodynamic parameters. However, as mentioned before, although we used an advanced hemodynamic monitoring system for study measurements, our approach does not allow drawing definite conclusions about the impact of RBC transfusion on oxygen extraction and oxygen transport. Therefore, larger studies on this subject allowing the determination of oxygen extraction calculated using oxygen consumption are needed in the future to learn more about the impact of RBC transfusion on oxygen transport. More data in this area are needed, to have a more differentiated view on transfusion triggers in critically ill patients.

### Limitations of the study

The low number of patients studied and the fact that we performed our study in a single ICU, are limitations of our study. Furthermore, the use of vasoactive drugs during RBC transfusion and study measurements might have influenced the results regarding CO.

## Conclusions

In ICU patients, transfusion of 2 RBC units induces a significant decrease in CO and CI because of a significant decrease in heart rate (while SV remains unchanged). Despite the decrease in CO, DO_2_ significantly increases because of a significant increase in CaO_2_. In addition, RBC transfusion results in a significant increase in MAP and SVRI. No significant changes in TPTD-parameters reflecting cardiac preload (GEDVI), pulmonary edema (EVLWI), and pulmonary vascular permeability (PVPI) are observed following RBC transfusion.

### Key messages

– Anemia is a common finding in intensive care unit patients and is usually treated with red blood cell transfusion. There are limited data on the impact of red blood cell transfusion on advanced hemodynamic parameters reflecting cardiopulmonary function.

– According to our results, in ICU patients, the transfusion of 2 red blood cell units induces a significant decrease in cardiac output and cardiac index because of a significant decrease in heart rate (while stroke volume remains unchanged). In addition, red blood cell transfusion results in a significant increase in mean arterial pressure and systemic vascular resistance index.

– Despite the decrease in cardiac output, oxygen delivery significantly increases because of a significant increase in arterial oxygen content.

– No significant changes in transpulmonary thermodilution-derived parameters reflecting cardiac preload, pulmonary edema, and pulmonary vascular permeability are observed following red blood cell transfusion.

## Abbreviations

CaO_2_: Arterial oxygen content; CI: Cardiac index; CO: Cardiac output; CPI: Cardiac power index; CVP: Central venous pressure; DO_2_: Oxygen delivery; dPmax: Index of left ventricular contractility; EVLWI: Extravascular lung water index; GEDVI: Global end-diastolic volume index; Hb: Hemoglobin concentration; ICU: Intensive care unit; MAP: Mean arterial pressure; PaCO_2_: Arterial partial pressure of carbon dioxide; PaO_2_: Arterial partial pressure of oxygen; PVPI: Pulmonary vascular permeability index; RBC: Red blood cell; SaO_2_: Arterial oxygen saturation; ScvO_2_: Central venous oxygen saturation; SV: Stroke volume; SvO_2_: Mixed venous oxygen saturation; SVRI: Systemic vascular resistance index; TPTD: Transpulmonary thermodilution; TRALI: Transfusion-related acute lung injury.

## Competing interests

Bernd Saugel and Wolfgang Huber collaborate with Pulsion Medical Systems (Munich, Germany) as members of the Medical Advisory Board. All other authors declare that they have no competing interests.

## Authors’ contributions

BS, VP, RMS, and WH conceived and designed the study. BS, MK, VP, and CS were responsible for acquisition of data. BS, AH, ASM, and WH performed the statistical analyses. BS, MK, VP, CS, ASM, MM, RMS, and WH were responsible for data analysis and interpretation. BS, AH, VP, CS, ASM, MM, and WH drafted the manuscript. RMS critically revised the manuscript for important intellectual content. RMS and WH supervised the study. All authors read and approved the final version of the manuscript.
